# Real-World Long-Term Clinical Outcomes of Ultrathin Strut Biodegradable Polymer Drug-Eluting Stents in Korean ST-Segment-Elevation Myocardial Infarction (STEMI) Patients with or without Acute Heart Failure Undergoing Primary Percutaneous Coronary Intervention

**DOI:** 10.3390/jcm10245898

**Published:** 2021-12-15

**Authors:** Albert Youngwoo Jang, Jongwook Yu, Pyung Chun Oh, Minsu Kim, Soon Yong Suh, Kyounghoon Lee, Seung Hwan Han, Woong Chol Kang

**Affiliations:** 1 Cardiology, Gachon University Gil Medical Center, Incheon 21565, Korea; cardio_gil@gilhospital.com (A.Y.J.); likemed@gilhospital.com (P.C.O.); msgene@gilhospital.com (M.K.); ssy@gilhospital.com (S.Y.S.); vancolee@gmail.com (K.L.); shhan@gilhospital.com (S.H.H.); 2 Department of Internal Medicine, Yonsei University College of Medicine, Seoul 03722, Korea; youjwjw@naver.com

**Keywords:** STEMI, ultrathin strut, biodegradable polymer, durable polymer, real-world, acute heart failure

## Abstract

Biodegradable polymers (BDPs) and ultrathin struts were recently introduced to drug-eluting stents (DES) to further improve outcomes. In this study, we analyzed and compared the effect of the ultrathin strut BDP-DES (UBDP-DES) with the conventional durable polymer-DES (DP-DES) in patients with ST-segment elevation myocardial infarction (STEMI) who underwent primary percutaneous coronary intervention (PPCI). A total of 356 STEMI patients (n = 160 in the UBDP-DES group and n = 196 in the DP-DES group) were enrolled. The primary endpoint was target lesion failure (TLF), including cardiac death, target vessel myocardial infarction, and ischemic-driven, target lesion revascularization (ID-TLR). The mean age was 60.3 ± 12.7 years (male 81.7%), and the median follow-up duration was 63.8 months. TLF was numerically more frequent in the UBDP-DES group (8.1% vs. 4.1%; HR 2.14; 95% CI 0.89–5.18; *p* = 0.091). Propensity score matching (PSM) was performed to balance discrepancies in the baseline characteristics due to patients in the UBDP-DES group initially having more unstable vital signs. However, after PSM (n = 116 in each group), there was no significant difference in TLF (5.3% vs. 5.3%; HR 1.04, 95% CI 0.34-3.22; *p* = 0.947) or other secondary endpoints including ID-TLR. In the subgroup analysis, subjects with initial acute heart failure (AHF), defined as Killip class ≥ 3, were associated with 13.6% chance of 30-day mortality (9-fold of those without AHF), although chances of repeat revascularization were low (3.0%). Among patients with AHF, the UBDP-DES group was associated with a numerically higher chance of TLF compared with the DP-DES group. There was no difference in TLF between groups in patients without AHF. This study showed that UBDP-DES has long-term clinical outcomes similar to those of conventional DP-DES in real-world Korean STEMI patients receiving PPCI, especially in those without initial AHF.

## 1. Introduction

Drug-eluting stents (DESs) have changed the landscape of percutaneous coronary intervention (PCI) by markedly reducing the rate of in-stent restenosis [1]. The greatest challenge with first-generation DESs is the high rate of stent thrombosis (ST) [2,3,4,5]. Durable polymers (DPs), substances coated around the stent struts to slowly elute anti-proliferative limus drugs, are thought to be one of the major causes of ST [4,6,7]. Once the drug is completely released, the DP no longer has function yet remains as a foreign substance inducing chronic inflammation and subsequent late/very late ST [8,9,10,11,12]. DESs using biodegradable polymers (BDPs) have been recently introduced as an attempt to reduce polymer-induced inflammation and ST, although the effect is still controversial [13,14,15,16,17,18,19]. The thickness of stent struts has also been suspected to be an important factor for outcomes. The first-generation DESs and bare metal stents (BMS) had thick stent struts, which were shown to cause turbulence and delay re-endothelialization [20]. Second-generation DESs using thinner struts have demonstrated superior outcomes over first-generation DESs. One of the recently introduced ultrathin strut DESs has shown promising results in previous trials, suggesting that a thinner strut thickness may potentially be responsible for improved outcomes [21]. The Orsiro™ (Biotronik AG, Bülach, Switzerland) stent adopts both the BDP and ultrathin struts, which have shown promising results [22,23]. Additionally, real-world data regarding the clinical characteristics and longitudinal outcomes of subjects with initial acute heart failure (AHF) among STEMI patients are limited. In this study, we sought to retrospectively compare the real-world long-term outcomes of the ultrathin strut BDP-DES (UBDP-DES), the Orsiro™ (Biotronik AG, Bülach, Switzerland) stent, and the durable polymer DESs (DP-DES), the Xience V™ (Abbott Vascular, Santa Clara, CA, USA) and Endeavor Resolute™ (Medtronic Vascular, Santa Rosa, CA, USA), in patients with or without AHF who underwent primary percutaneous coronary intervention (PPCI) due to ST-segment elevation myocardial infarction (STEMI).

## 2. Materials and Methods

### 2.1. Study Design and Patient Selection

**Figure 1:**
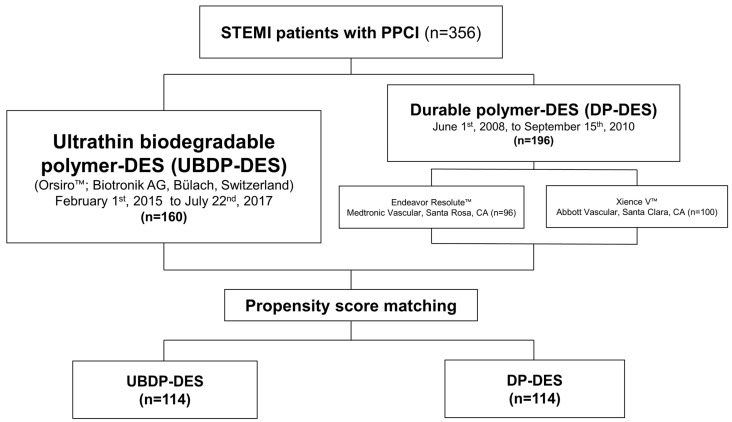
Diagram of the study design.

This was a single-center, prospective, observational study. STEMI patients aged 20 years or older who underwent PPCI between June 2008 and July 2017 were enrolled. STEMI was diagnosed based on clinical findings, such as chest pain, 12-lead electrocardiography findings, and cardiac markers in the emergency room (ER) setting. The Institutional Review Board of Gachon University Gil Medical Center approved this study (GDIRB2021-267), which complies with the Declaration of Helsinki (6th revision). All patients provided written informed consent prior to enrollment. The exclusion criteria were as follows: thrombolytic therapy; history of allergy or anaphylaxis reaction to antiplatelet agents, steel, or heparin; history of cardiomyopathy or valvulopathy (≥moderate). A total of 356 consecutive patients with STEMI who underwent PPCI were enrolled in this study (Figure 1). AHF was defined as Killip class ≥ 3.

### 2.2. Primary PPCI

All procedures were performed according to current standard guidelines. Before PPCI, patients were pre-medicated with aspirin (at least 100 mg) and a loading dose of P2Y12 receptor antagonist. Heparin was administered throughout the PPCI to maintain an activated clotting time of ≥250 s. A glycoprotein IIb/IIIa receptor blocker was administered at the operator’s discretion. Coronary angiography was performed using the standard techniques. Decisions to use thrombectomy devices, intravascular ultrasound, an intra-aortic balloon pump, or percutaneous cardiopulmonary support were made by the operator. STEMI patients implanted with the UBDP-DES (Orsiro™ (Biotronik AG, Bülach, Switzerland); n = 160; from 1 February 2015 to 22 July 2017) were compared to our historic cohort implanted with the DP-DES (Xience V™ [Abbott Vascular, Santa Clara, CA, USA) and Endeavor Resolute™ (Medtronic Vascular, Santa Rosa, CA, USA); n = 196; from 1 June 2008 to 15 September 2010).

### 2.3. Follow Up

After PPCI, all patients were monitored in a coronary care unit for at least 24 h. Two-dimensional transthoracic echocardiography was performed within 24 h of the index procedure. Standard medical management, including dual antiplatelet agents, beta blockers, statins, nitrates, angiotensinogen converting enzyme inhibitors, or angiotensin II receptor blockers, was provided by the designated physicians. After discharge, the patients were regularly followed up at outpatient clinics. All medical records were reviewed to assess the outcomes.

### 2.4. Study Endpoint

The primary endpoint was target lesion failure (TLF), defined as a composite of cardiac death, non-fatal target vessel myocardial infarction (TVMI), and ischemic-driven target lesion revascularization (ID-TLR). Secondary endpoints were individual constituents of the primary endpoint, stroke, all-cause mortality, and stent thrombosis (ST).

### 2.5. Statistical Analysis

Continuous variables are presented as means ± standard deviations for normally distributed data or as medians (interquartile ranges) for skewed data. Intergroup differences were determined using Student’s *t*-test or the Mann–Whitney U test. The normality of variables was evaluated using the Shapiro–Wilk test (*p* > 0.05, normal). Categorical variables are expressed as absolute numbers and percentages and were analyzed using the chi-square test or Fisher’s exact test. Two-sided *p*-values of <0.05 were considered statistically significant.

Since significant imbalances such as more unstable initial vital signs in the UBDP-DES group were observed, propensity score matching (PSM) was performed. Propensity scores for each group were calculated using logistic regression analysis. The two groups were matched for 14 pre-procedural clinical and angiographic parameters (type of DES stent (UBDP-DES or DP-DES), N-terminal pro-brain natriuretic peptide (NT-proBNP), troponin I, high-sensitivity C-reactive protein (hs-CRP), cardiopulmonary resuscitation (CPR), body mass index, sex, age, HbA1c, a history of hypertension or stroke, Killip class ≥ 3, pre-procedural thrombolysis in myocardial infarction (TIMI) flow ≥ 3, and left ventricular ejection fraction (LVEF)). Both groups were matched one-to-one with a caliper width of 0.1, using the nearest neighbor method. A standard mean difference less than 0.1 after PSM is considered adequately balance between baseline covariates.

Longitudinal survival data, with or without PSM, were plotted using Kaplan–Meier plots. Predictor analysis was performed using a stepwise Cox proportional hazards regression model. Adjusted hazard ratios (HRs) and 95% confidence intervals (CIs) after multivariable adjustment were calculated for each stent group. Covariates that either were statistically significant in the univariate Cox analysis or had clinical relevance were included in the multivariable Cox analysis. The analysis was performed using SPSS (version 23.0; IBM Corp., Armonk, NY, USA) and R Statistical Software (version 3.6.0; R Foundation for Statistical Computing, Vienna, Austria).

## 3. Results

### 3.1. Baseline Characteristics

A total of 356 patients (81.7% male, mean age 60.3 ± 12.7 years) were enrolled and followed up for a median of 63.8 months. UBDP-DES and DP-DES were implanted in 160 and 196 patients, respectively (Figure 1), and 3.4%, 18.5%, and 7.5% of patients initially presented with shock, Killip class ≥ 3, and CPR, respectively. There was no significant difference in demographic data except for BMI between the DP-DES and UBDP-DES groups (Table 1). However, the vital signs of the UBDP-DES group appeared to be more unstable than those of the DP-DES group, as the presence of shock, Killip class ≥ 3, or CPR at presentation was significantly more frequent in the UBDP-DES group. PSM was performed to balance the discrepancies between the two groups. After PSM, 114 patients were allocated to each stent group (Figure 1). The baseline laboratory results are presented in Table 1. The initial NT-proBNP and hs-CRP levels were higher in the DP-DES group in the pre-PSM analysis. Peak levels of troponin-I was higher in the DP-DES group, while there was no difference in peak creatinine kinase myocardial band (CK-MB) or NT-proBNP. Overall, there was no significant difference in demographic data, vital signs, or laboratory findings between the two groups (Table 1).

### 3.2. Coronary and Procedural-Related Characteristics

There was no significant difference in the extent of coronary artery disease (CAD) or location of the infarct-related artery (IRA) between groups in the pre-PSM analysis (Table 2). Pre-PCI TIMI flow≥ 3 was more frequently observed in the DP-DES group. After matching, there was no significant difference in the extent of CAD, location of IRA, and pre-PCI TIMI ≥3 flow (Table 2).

### 3.3. Discharge Medication

The discharge medications are shown in Table 2. Since the historic control group, the DP-DES group, was before prasugrel (2010) or ticagrelor (2016) were available in Korea, there were no patients prescribed with such medication. The use of statins was less prevalent in the DP-DES group pre- and post-PSM (Table 3). Discharge medications were not included in the PSM process because they were decided after the index PPCI.

### 3.4. Outcome Analysis

**Figure 2:**
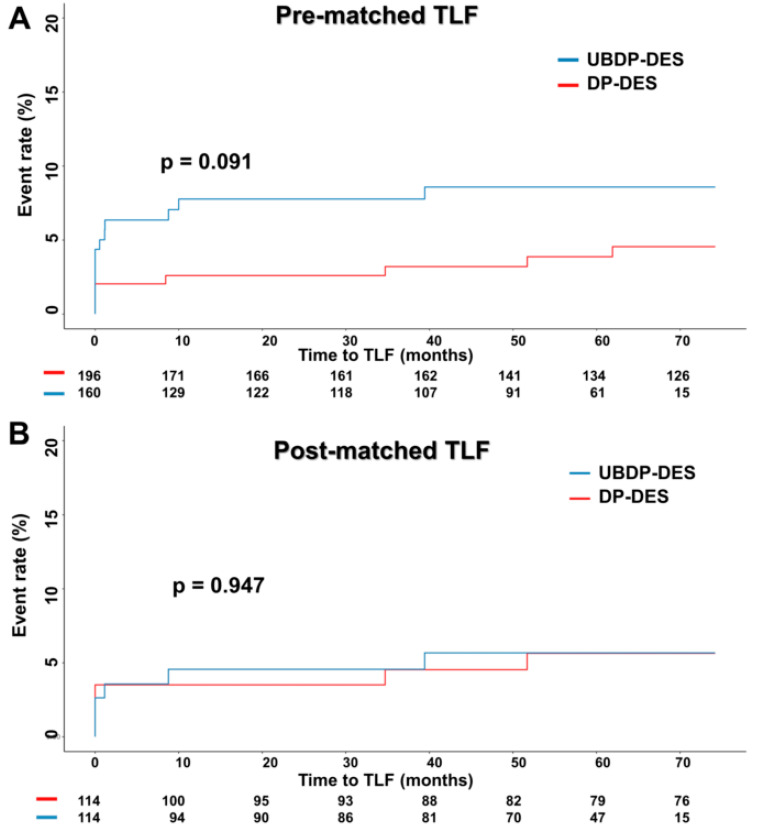
Kaplan–Meier survival curves of TLF (**A**) pre- and (**B**) post-PSM. All abbreviations are listed in Table 1.

**Figure 3:**
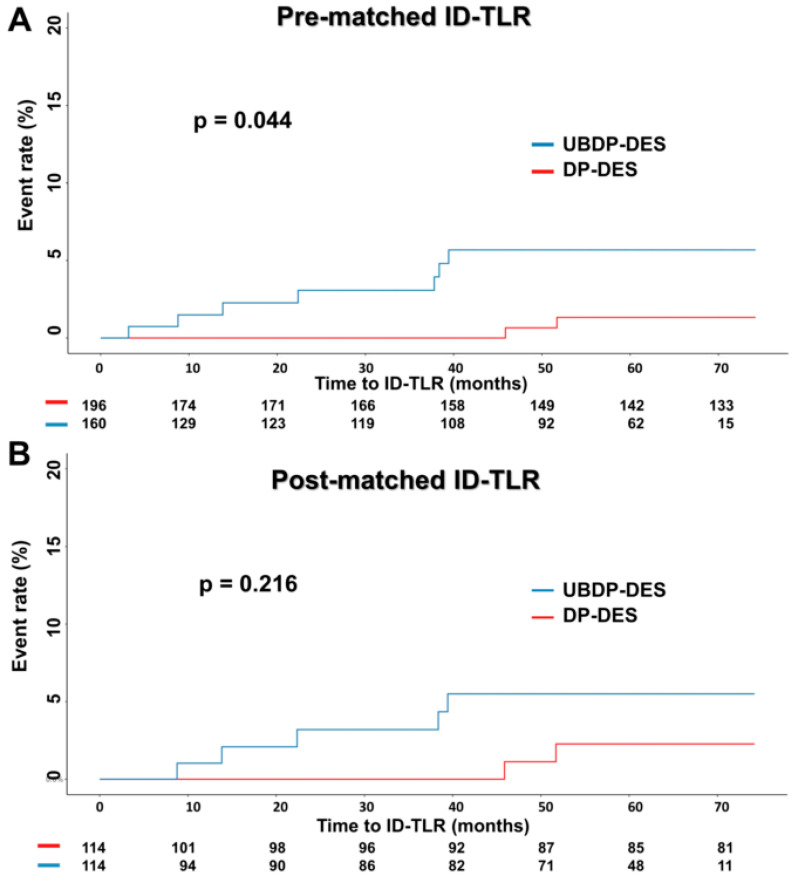
Kaplan–Meier survival curves of ID-TLR (**A**) pre- and (**B**) post-PSM. All abbreviations are listed in Table 1.

In the crude analysis, ID-TLR and TVR were significantly more frequent in the UBDP-DES group (Table 4). The UBDP-DES group showed a strong trend of more cardiac deaths and 30-day in-hospital mortalities. The number of events among all endpoints was similar after PSM (Table 4). The longitudinal outcome analysis is presented in Table 5. In the pre-PSM longitudinal analysis, the UBDP-DES group was associated with a significantly higher rate of ID-TLR. The UBDP-DES also had a quantitatively higher rate of cardiac death and MACEs pre-PSM. Multivariate Cox regression and PSM analyses were performed to adjust for the discrepancies in the baseline characteristics shown in Table 1 and Table 2. ID-TLR and TVR remained significantly higher in the UBDP-DES group after multivariate adjustment. However, after PSM, all the primary and secondary endpoints were similar (Table 5). Kaplan–Meier plots of pre- and post-PSM MACE and ID-TLR are shown in Figure 2 and Figure 3, respectively.

### 3.5. Subgroup Analysis

**Figure 4:**
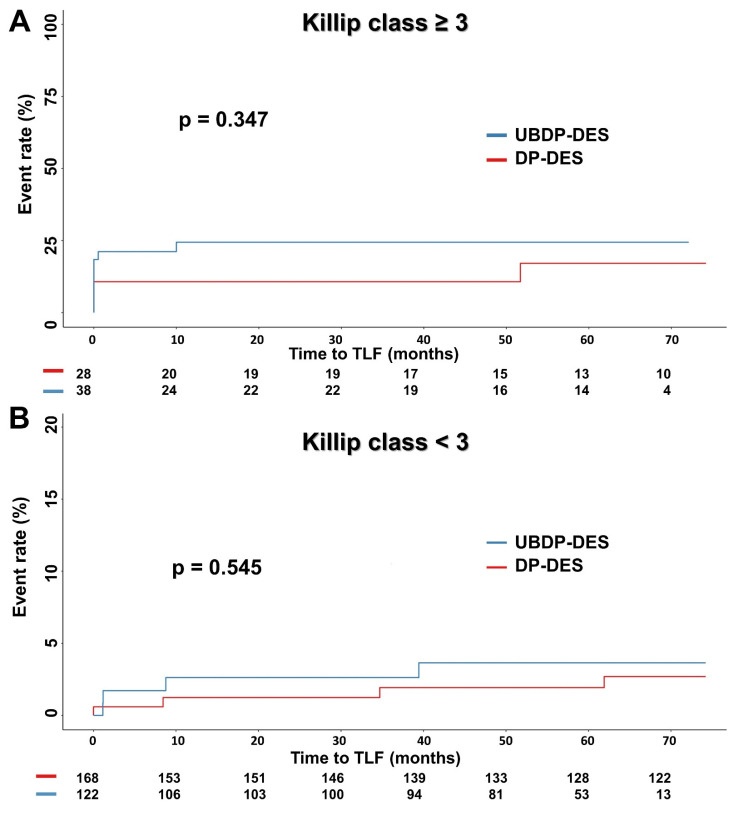
Kaplan–Meier survival curves of TLF in subjects presenting (**A**) with or (**B**) without Killip class ≥ 3.

We further analyzed pre-PSM baseline characteristics (Supplementary Table S1) and longitudinal outcomes in patients presenting with or without AHF, which was defined as Killip class ≥ 3 (Figure 4). The 30-day mortality in those with AHF was 13.6%, which was 9-fold of those without AHF. Cardiac death was the cause of majority of deaths (92.8%), althoutg repeat revascularization, including TVMI and ID-TLR, was only 3.0%. This suggests that most mortalities were related to heart failure (Supplementary Table S1). The proportion of patients presenting with initial CPR were more than 3-fold in the UBDP-DES group compared with the DP-DES group, suggesting that patients in the UBDP-DES group were more severe in clinical status. The UBDP-DES group was associated with numerically more TLFs in patients presenting with AHF (Figure 4A). However, there was no difference in TLF in those with Killip class < 3 (Figure 4B). The pre-PSM survival analysis after excluding patients who died during the first admission are presented in Supplementary Figure S1. There was no difference in TLF between groups.

## 4. Discussion

In the crude analysis, the UBDP-DES group had significantly higher rates of ID-TLR and TVR and strong trends of a higher risk of cardiac death and MACE. Since the UBDP-DES group appeared to have more severe patients enrolled at baseline—such as more patients presenting with hypotension, pulmonary edema, lower LVEF, and more CPR at presentation—we performed PSM to balance these discrepancies. After PSM, there were no differences in primary or secondary endpoints. The 30-day mortality in Korean STEMI patients presenting with initial AHF was high (13.6%), although the chances repeat revascularization, TVMI or ID-TLR, were low (3.0%). In this study, we found that the UBDP-DES group had similar composite clinical outcomes to the DP-DES group in patients receiving PPCI due to STEMI.

Recently, the BIOSTEMI (BDP sirolimus-eluting stents versus durable polymer everolimus-eluting stents in patients with STEMI) trial showed that the UBDP-DES had superior results in terms of composite endpoint driven by lower rates of ID-TLR [23]. These results were consistent in a meta-analysis comparing UBDP-DES to second generation DP-DESs. The UBDP-DES showed superior results in terms of TLF with an absolute risk reduction of 1.3%, mainly driven by reduced TVMI [21].

Our crude analysis demonstrated results opposite to those of the BIOSTEMI trial. Even after PSM, we were not able to demonstrate superior results in the UBDP-DES group. Our findings may be attributed to several factors. First, the difference in standard clinical practice during the period of DP-DES control and the UBDP-DES group may have caused the discrepancy in data. The DP-DES group was enrolled in the late 2000s, whereas the UBDP-DES group was enrolled in the mid-2010s. In the late 2000s, the Korean government implemented a policy to promote PPCI within 60 min in STEMI patients presenting to the ER [24], which was deregulated several years later in the mid-2010s. Most of the patients in the DP-DES group were enrolled during the period of mandatory PPCI within 60 min, while the UBDP-DES patients were registered after the regulations were alleviated. Differences in clinical practice may have confounded these outcomes. Secondly, the real-world evidence does not necessarily reflect randomized control trials (RCTs). Many factors can confound the outcomes of real-world practice [25].

Patients enrolled in the registries appear to be more severe than those in the RCTs, which may, at least in part, account for the discrepancies with major RCTs. Although the BIOSTEMI trial did not present the severity of symptoms or vital signs, recent RCTs included subjects with relatively less severe symptoms or vital signs. The patients enrolled in the latest SAFARI-STEMI (Safety and Efficacy of Femoral Access vs. Radial Access in ST-Elevation Myocardial Infarction) [26] and TOTAL (Trial of Routine Aspiration Thrombectomy with PCI versus PCI Alone in Patients with STEMI) study had less severe Killip classes and vital signs [27]. For example, subjects with Killip class ≥ 3 comprised only 1.0%, and the systolic blood pressure was approximately 10 mmHg higher than our study in the SAFARI-STEMI trial. In contrast, the proportion of patients with shock or pulmonary edema was remarkably higher in recent registries. In the INTERSTELLAR (INcheon-Bucheon cohorT of patients undERgoing primary percutaneous coronary intervention for acute ST-ELevation myocardiaL infARction) registry, the percentage of those presenting with Killip class ≥ 3 was as high as 20%, depending on the subgroup [28,29]. A large Swedish registry showed that the percentage of patients presenting with cardiac arrest was 6.5% [30]. These discrepancies in severity of patients between RCTs and registries may translate to different clinical outcomes of the stents.

The effect of ultrathin struts on clinical outcomes is controversial. The LEADERS (Limus Eluted From A Durable Versus ERodable Stent Coating) trial was the first trial to compare the outcome between BDP-DES and DP-DES [13]. The LEADERS trial demonstrated that the BDP-DES was noninferior to the first-generation sirolimus-eluting stent with durable polymers, although late ST at 9 and 12 months was significantly lower in the BDP-DES group. The COMPARE II (Comparison of the Everolimus Eluting With the Biolimus A9 Eluting Stent) and NEXT (Nobori Biolimus-eluting Versus Xience/Promus Everolimus-eluting Stent Trial) studies compared BDP-DESs to DP-DESs [14,15]. These studies showed that BDP-DESs were noninferior to DP-DES. The most recent study is the HOST-REDUCE-POLYTECH-ACS (Harmonizing Optimal Strategy for Treatment of Coronary Artery Diseases—Comparison of Reduction of Prasugrel Dose or Polymer Technology in ACS Patients) trial comparing the outcomes of second-generation BDP-DES versus DP-DES in patients with acute coronary syndrome [31]. In this study, the DP-DES showed noninferiority to BDP-DES. Overall, it appears that BDP-DES and DP-DES have similar outcomes.

The superior outcomes of the UBDP-DES compared with the DP-DES in the BIOSTEMI may be attributed to either the BDP or ultrathinness of the struts [21,23]. The previously mentioned trials (LEADERS, COMPARE II, NEXT, and HOST-REDUCE-POLYTECH-ACS) demonstrated that BDPs may not dramatically improve outcomes compared with DP-DESs. Additionally, a meta-analysis demonstrated that newer-generation ultrathin strut DESs improve outcomes [21]. These findings suggest that the superior outcomes of UBDP-DES compared with DP-DES in the BIOSTEMI trial may be due to the ultrathinness of stent struts more so than the BDP. Our study demonstrates that even in real-world practice, the UBDP-DES showed outcomes comparable with DP-DESs despite the more severe patients in the DP-DES group.

There are several limitations to this study. First, each stent group was enrolled during different periods of time. The potential change in clinical practice may have confounded the results, such as different door-to-balloon times or differences in antiplatelet therapy. Our study also has the intrinsic limitations of a single-center observational study, such as the small sample size. However, the consistency in medical staff and interventionists may also translate to the strength of the current investigation. In addition, the genetic homogeneity of patients and the associated metabolic characteristics may account for the discrepancy in results compared with the RCTs. Patients enrolled in this study were all Korean descendants [32], who may significantly differ in genetic traits and diet compared with Caucasians who were the majority of enrolled subjects in the BIOSTEMI trial [23].

## 5. Conclusions

This study showed that the UBDP-DES has similar long-term clinical outcomes to those of the conventional DP-DES in STEMI patients receiving PPCI, especially in those without AHF. The 30-day mortality of STEMI patients presenting with AHF was 9-fold higher compared with those without AHF.

## Figures and Tables

**Table 1:** Baseline demographic, vital signs, and laboratory findings pre- and post-propensity score matching (PSM).

	Pre-PSM				Post-PSM			
	DP-DES (N = 196)	UBDP-DES (N = 160)	*p*	SMD	DP-DES (N = 114)	UBDP-DES (N = 114)	*p*	SMD
**Demographics**								
Male, n (%)	156 (79.6)	135 (84.4)	0.306	0.125	94 (82.5)	93 (81.6)	1.000	0.023
Age (years)	59.9 ± 12.9	60.8 ± 12.5	0.506	0.071	59.9 ± 12.2	60.3 ± 11.9	0.767	0.039
Height (cm)	166.8 ± 8.0	167.5 ± 7.8	0.477	0.094	166.9 ± 8.2	167.4 ± 7.9	0.643	0.061
Weight (kg)	67.1 ± 9.7	68.2 ± 9.6	0.382	0.115	68.3 ± 11.7	67.8 ± 9.4	0.738	0.044
BMI (kg/m²)	24.5 ± 3.0	23.8 ± 2.8	0.028	0.234	24.4 ± 3.1	24.2 ± 2.6	0.457	0.099
Current smoker, n (%)	109 (55.6)	92 (57.5)	0.803	0.038	70 (61.4)	64 (56.1)	0.501	0.107
HTN, n (%)	83 (42.3)	64 (40.0)	0.734	0.048	46 (40.4)	46 (40.4)	1.000	<0.001
DM, n (%)	46 (23.5)	37 (23.1)	1.000	0.008	25 (21.9)	25 (21.9)	1.000	<0.001
CHF, n (%)	5 (2.6)	2 (1.2)	0.465	0.095	5 (4.4)	2 (1.8)	0.446	0.153
MI, n (%)	6 (3.1)	3 (1.9)	0.523	0.077	4 (3.5)	3 (2.6)	1.000	0.051
PCI, n (%)	6 (3.1)	8 (5.0)	0.508	0.099	3 (2.6)	6 (5.3)	0.499	0.135
CABG, n (%)	0 (0.0)	0 (0.0)	NA	<0.001	0 (0.0)	0 (0.0)	NA	<0.001
Stroke, n (%)	5 (2.6)	7 (4.4)	0.514	0.100	4 (3.5)	4 (3.5)	1.000	<0.001
ESRD, n (%)	1 (0.5)	2 (1.2)	0.590		0 (0.0)	0 (0.0)	NA	<0.001
**Vital Signs**								
SBP (mmHg)	129.1 ± 25.2	130.1 ± 31.8	0.795	0.034	130.1 ± 26.4	130.9 ± 29.5	0.844	0.026
DBP (mmHg)	79.6 ± 17.5	77.9 ± 19.5	0.473	0.094	80.2 ± 17.2	79.6 ± 18.3	0.786	0.036
Heart rate (beats/minute)	78.1 ± 17.6	77.8 ± 20.2	0.917	0.014	78.8 ± 18.1	78.1 ± 19.8	0.796	0.034
Shock, n (%)	1 (0.5)	11 (6.9)	0.003	0.342	1 (0.9)	3 (2.6)	0.622	0.134
Killip class ≥ 3, n (%)	28 (14.3)	38 (23.8)	0.032	0.243	17 (14.9)	14 (12.3)	0.699	0.077
CPR at initial presentation, n (%)	8 (4.1)	19 (11.9)	0.010	0.291	7 (6.1)	2 (1.8)	0.171	0.227
LVEF (%)	50.1 ± 11.7	45.5 ± 12.8	0.001	0.374	46.9 ± 11.0	47.9 ± 11.6	0.515	0.086
**Laboratory Findings**								
Hb (mg/dL)	14.4 ± 1.8	14.6 ± 1.9	0.275	0.117	14.5 ± 1.6	14.6 ± 1.9	0.852	0.025
Glucose (mg/dL)	171.0 ± 71.4	180.4 ± 84.5	0.264	0.120	168.1 ± 60.8	172.9 ± 75.3	0.590	0.071
HbA1c (%)	6.3 ± 1.8	6.3 ± 1.5	0.966	0.005	6.3 ± 1.8	6.2 ± 1.4	0.904	0.016
LDL-C (mg/dL)	114.7 ± 36.6	110.2 ± 37.2	0.255	0.122	117.3 ± 37.9	108.9 ± 35.7	0.086	0.229
Creatinine (mg/dL)	1.1 ± 0.7	1.2 ± 0.7	0.273	0.117	1.0 ± 0.5	1.1 ± 0.7	0.238	0.157
Total protein (mg/dL)	7.1 ± 0.6	7.4 ± 4.0	0.347	0.105	7.1 ± 0.6	7.2 ± 0.6	0.302	0.137
Albumin (g/dL)	4.1 ± 0.3	4.1 ± 0.4	0.728	0.037	4.1 ± 0.3	4.1 ± 0.4	0.970	0.005
Total bilirubin (g/dL)	0.7 ± 0.4	0.7 ± 0.3	0.078	0.186	0.7 ± 0.4	0.7 ± 0.3	0.988	0.002
AST (U/L)	60.3 ± 82.4	65.4 ± 74.4	0.541	0.065	58.8 ± 84.7	65.9 ± 78.5	0.511	0.087
ALT (U/L)	34.2 ± 28.2	38.6 ± 36.3	0.203	0.138	32.5 ± 24.8	36.6 ± 27.8	0.238	0.157
Uric acid (mg/dL)	5.8 ± 1.6	5.9 ± 1.8	0.940	0.008	5.8 ± 1.6	5.7 ± 1.9	0.723	0.047
CPK (U/L)	546.4 ± 880.9	405.4 ± 660.2	0.086	0.181	572.0 ± 931.0	465.9 ± 744.4	0.343	0.126
Initial NT-proBNP (pg/mL [IQR])	342.0 [43.2–2810.5]	156.5 [39.0–809.0]	0.031	0.114	289.9 [20.6, 2281.1]	156.5 [36.2, 1051.2]	0.318	0.077
Peak NT-proBNP (pg/mL [IQR])	140.6 [73.4–279.5]	220.5 [62.2–300.0]	0.031	0.265	201.6 [106.1–300.0]	212.0 [64.0–300.0]	0.825	0.023
Initial CK-MB (ng/mL)	31.5 ± 65.2	27.5 ± 59.8	0.547	0.064	32.2 ± 68.2	32.6 ± 67.2	0.968	0.005
Peak CK-MB (ng/mL)	162.0 ± 102.6	189.6 ± 117.3	0.019	0.218	192.0 ± 101.0	186.6 ± 115.0	0.704	0.035
Initial Troponin I (ng/mL)	7.9 ± 15.8	6.1 ± 13.7	0.260	0.119	7.1 ± 14.9	7.4 ± 15.1	0.899	0.017
Peak Troponin I (ng/mL)	43.4 ± 14.2	41.7 ± 15.1	0.278	0.117	45.6 ± 11.9	42.3 ± 15.0	0.069	0.350
Initial hs-CRP (mg/dL)	1.7 ± 3.1	0.6 ± 2.1	<0.001	0.380	1.1 ± 2.4	0.8 ± 2.5	0.317	0.133

DP-DES, durable polymer drug-eluting stent; UBDP-DES, ultrathin strut biodegradable polymer drug-eluting stent; SMD, standard mean difference; BMI, body mass index; HTN, hypertension; DM, diabetes mellitus; CHF, congestive heart failure; MI, myocardial infarction; PCI, percutaneous coronary intervention; CABG, coronary artery bypass graft surgery; ESRD, end-stand renal disease; SBP, systolic blood pressure; DBP, diastolic blood pressure; CPR, cardiopulmonary resuscitation; LVEF, left ventricular ejection fraction; Hb, hemoglobin; LDL-C, low-density lipoprotein cholesterol; AST, aspartate transaminase; ALT, alanine transaminase; CPK, creatine phosphate kinase; CK-MB, creatinine kinase myocardial band; NT-proBNP, N-terminal pro-B-type natriuretic peptide; hsCRP, high-sensitivity C-reactive protein; NA, not applicable; SMD, standard mean difference.

**Table 2:** Baseline coronary and procedure-related characteristics pre- and post-PSM.

	Pre-PSM				Post-PSM			
	DP-DES (N = 196)	UBDP-DES (N = 160)	*p*	SMD	DP-DES (N = 114)	UBDP-DES (N = 114)	*p*	SMD
**Coronary Characteristics**								
Extent of CAD			0.406	0.144			0.610	0.132
1-VD, n (%)	73 (37.2)	49 (30.6)			40 (35.1)	33 (28.9)		
2-VD, n (%)	70 (35.7)	61 (38.1)			43 (37.7)	47 (41.2)		
3-VD, n (%)	53 (27.0)	50 (31.2)			31 (27.2)	34 (29.8)		
Infarct-related artery			0.218	0.244			0.741	0.175
LM, n (%)	2 (1.0)	0 (0.0)			1 (0.9)	0 (0.0)		
LAD, n (%)	98 (50.0)	95 (59.4)			66 (57.9)	61 (53.5)		
LCX, n (%)	7 (3.6)	7 (4.4)			3 (2.6)	6 (5.3)		
RCA, n (%)	89 (45.4)	58 (36.2)			43 (37.7)	47 (41.2)		
Lesion characteristics								
CTO, n (%)	0 (0.0)	0 (0.0)	NA	<0.001	0 (0.0)	0 (0.0)	NA	<0.001
Severe calcification, n (%)	5 (2.6)	7 (4.4)	0.514	0.1	4 (3.5)	4 (3.5)	1.000	<0.001
**Procedure-related Characteristics**								
Baseline TIMI flow, n (%)			<0.001	0.550			0.025	0.432
TIMI 0, n (%)	105 (53.6)	80 (50.0)			70 (61.4)	61 (53.5)		
TIMI 1, n (%)	25 (12.8)	14 (8.8)			15 (13.2)	8 (7.0)		
TIMI 2, n (%)	35 (17.9)	59 (36.9)			20 (17.5)	39 (34.2)		
TIMI 3, n (%)	30 (15.3)	7 (4.4)			8 (7.0)	6 (5.3)		
Pre-PCI TIMI ≥ 3, n (%)	30 (15.3)	7 (4.4)	<0.001	0.390	8 (7.0)	6 (5.3)	0.603	0.153
Number of stents	1.16 ± 0.41	1.10 ± 0.30	0.031	0.063	1.18 ± 0.45	1.10 ± 0.31	0.125	0.079
Stent diameter (mm)	3.1 ± 0.4	3.1 ± 0.4	0.876	0.021	3.1 ± 0.4	3.0 ± 0.4	0.108	0.214
Stent length (mm)	28.8 ± 10.3	25.9 ± 9.8	0.031	0.284	29.4 ± 11.5	26.4 ± 9.7	0.033	0.284
Final TIMI flow, n (%)			0.067	0.275			0.076	0.332
TIMI 0, n (%)	6 (3.1)	0 (0.0)			0 (0.0)	0 (0.0)		
TIMI 1, n (%)	11 (5.6)	8 (5.0)			3 (2.6)	0 (0.0)		
TIMI 2, n (%)	1 (0.5)	0 (0.0)			7 (6.1)	8 (7.0)		
TIMI 3, n (%)	178 (90.8)	152 (95.0)			104 (90.4)	106 (93.0)		
Post-PCI TIMI ≥ 3, n (%)	178 (90.8)	152 (95.0)	0.220	0.179	103 (90.4)	106 (93.0)	0.622	0.149

CAD, coronary artery disease; VD, vessel disease; LM, left main; LAD, left anterior descending artery; LCX, left circumflex artery; RCA, right coronary artery; CTO, chronic total occlusive disease; TIMI, thrombolysis in myocardial infarction; SMD, standard mean difference. All other abbreviations are listed in Table 1.

**Table 3:** Discharge medications pre- and post-PSM.

	Pre-PSM				Post-PSM			
	DP-DES (N = 196)	UBDP-DES (N = 160)	*p*	SMD	DP-DES (N = 114)	UBDP-DES (N = 114)	*p*	SMD
**Discharge Medication**								
Aspirin, n (%)	196 (100.0)	160 (100.0)	NA	1.000	114 (100.0)	113 (99.1)	NA	1.000
Clopidogrel, n (%)	196 (100.0)	30 (18.8)	<0.001	2.944	114 (100.0)	24 (21.1)	<0.001	2.739
Ticagrelor, n (%)	0 (0.0)	128 (80.0)	<0.001	2.828	0 (0.0)	89 (78.1)	<0.001	2.668
Prasugrel, n (%)	0 (0.0)	2 (1.2)	0.201	0.159	0 (0.0)	1 (0.9)	1.000	0.133
Beta-blocker, n (%)	184 (93.9)	140 (87.5)	0.057	0.221	106 (93.0)	103 (90.4)	0.632	0.095
ACEi/ARB, n (%)	174 (88.8)	132 (82.5)	0.123	0.180	97 (85.1)	95 (83.3)	0.856	0.048
Statin, n (%)	129 (65.8)	152 (95.0)	<0.001	0.791	77 (67.5)	110 (96.5)	<0.001	0.814

ACEi, angiotensin converting enzyme inhibitor; ARB, angiotensin receptor blocker; All other abbreviations are listed in Table 1.

**Table 4:** Clinical events pre- and post-PSM.

	Pre-PSM			Post-PSM		
	DP-DES (N = 196)	UBDP-DES (N = 160)	*p*	DP-DES (N = 114)	UBDP-DES (N = 114)	*p*
Median follow-up [months (IQR)]	74.2 [0.0, 74.2]	52.2 [0.0, 74.2]	<0.001	74.2 [45.3, 74.2]	53.4 [31.5, 66.0]	<0.001
All-cause mortality, n (%)	10 (5.1)	13 (8.1)	0.248	7 (6.1)	5 (4.4)	0.767
Cardiac death, n (%)	6 (3.1)	11 (6.9)	0.093	5 (4.4)	4 (3.5)	1.000
Non cardiac death, n (%)	4 (2.0)	2 (1.2)	0.564	2 (1.8)	1 (0.9)	1.000
30-day mortality, n (%)	3 (1.5)	7 (4.4)	0.120	3 (2.6)	3 (2.6)	1.000
TVMI, n (%)	2 (1.0)	2 (1.3)	1.000	1 (0.9)	2 (1.8)	1.000
ID-TLR, n (%)	2 (1.0)	7 (4.4)	0.045	2 (1.8)	5 (4.4)	0.446
Stent thrombosis, n (%)	2 (1.0)	2 (1.3)	1.000	1 (0.9)	2 (1.8)	1.000
Stroke, n (%)	10 (5.1)	5 (3.1)	0.356	8 (7.0)	3 (2.6)	0.216
TLF, n (%)	8 (4.1)	13 (8.1)	0.118	6 (5.3)	6 (5.3)	1.000

TVMI, target vessel myocardial infarction; ID-TLR, ischemic driven target lesion revascularization; TLF, target lesion failure. All other abbreviations are listed in Table 1.

**Table 5:** Clinical events with multivariable adjustment and pre-/post-PSM using Cox regression.

	DP-DES	UBDP-DES	HR (95% CI)	*p*
All-cause mortality				
Pre-matched unadjusted	10 (5.1)	13 (8.1)	1.86 (0.81–4.28)	0.144
Multivariable adjusted *	-	-	1.57 (0.54–4.53)	0.408
Propensity score matched	7 (6.1)	5 (4.4)	0.75 (0.24–2.35)	0.616
Cardiac death				
Pre-matched unadjusted	6 (3.1)	11 (6.9)	2.43 (0.89–6.58)	0.082
Multivariable adjusted *	-	-	2.49 (0.57–10.85)	0.225
Propensity score matched	5 (4.4)	4 (3.5)	0.82 (0.22–3.07)	0.773
TVMI				
Pre-matched unadjusted	2 (1.0)	2 (1.3)	1.35 (0.19–9.59)	0.764
Multivariable adjusted *	-	-	1.06 (0.09–12.40)	0.961
Propensity score matched	1 (0.9)	2 (1.8)	2.17 (0.20–23.93)	0.527
ID-TLR				
Pre-matched unadjusted	2 (1.0)	7 (4.4)	5.02 (1.04–24.21)	0.044
Multivariable adjusted *	-	-	13.97 (1.55–126.13)	0.019
Propensity score matched	2 (1.8)	5 (4.4)	2.82 (0.55–14.56)	0.216
Stroke				
Pre-matched unadjusted	10 (5.1)	5 (3.1)	0.80 (0.27–2.41)	0.690
Multivariable adjusted *	-	-	0.83 (0.25–2.77)	0.842
Propensity score matched	8 (7.0)	3 (2.6)	0.50 (0.13–1.95)	0.316
TLF				
Pre-matched unadjusted	8 (4.1)	13 (8.1)	2.14 (0.89–5.18)	0.091
Multivariable adjusted *	-	-	1.86 (0.60–5.76)	0.286
Propensity score matched	6 (5.3)	6 (5.3)	1.04 (0.34–3.22)	0.947

All abbreviations are listed in Table 1 and Table 3; * Adjusted for type of DES stent, NT-proBNP, troponin I, hs-CRP, CPR, body mass index, sex, age, HbA1c, a history of hypertension or stroke, Killip class ≥ 3, TIMI flow ≥ 3, and LVEF.

## Data Availability

Data is not available due to strict patient health information regulations.

## Supplementary Materials

The following are available online at https://www.mdpi.com/article/10.3390/jcm10245898/s1, Figure S1: The survival analysis after excluding patients with hospital mortality, Table S1: Clinical characteristics and outcome of patients presenting with Killip class ≥3.
