# Supplementary material for: Real-World Long-Term Clinical Outcomes of Ultrathin Strut Biodegradable Polymer Drug-Eluting Stents in Korean ST-Segment-Elevation Myocardial Infarction (STEMI) Patients with or without Acute Heart Failure Undergoing Primary Percutaneous Coronary Intervention
Source: J Clin Med. 2021 Dec 15;10(24):5898. doi: 10.3390/jcm10245898 (PMC8708844; doi:10.3390/jcm10245898)
Supplement: Supplementary file 1 [file jcm-10-05898-s001.zip › jcm-1501192-supplementary.pdf]

Supplementary Figure S1. The survival analysis after excluding patients with hospital mortality.

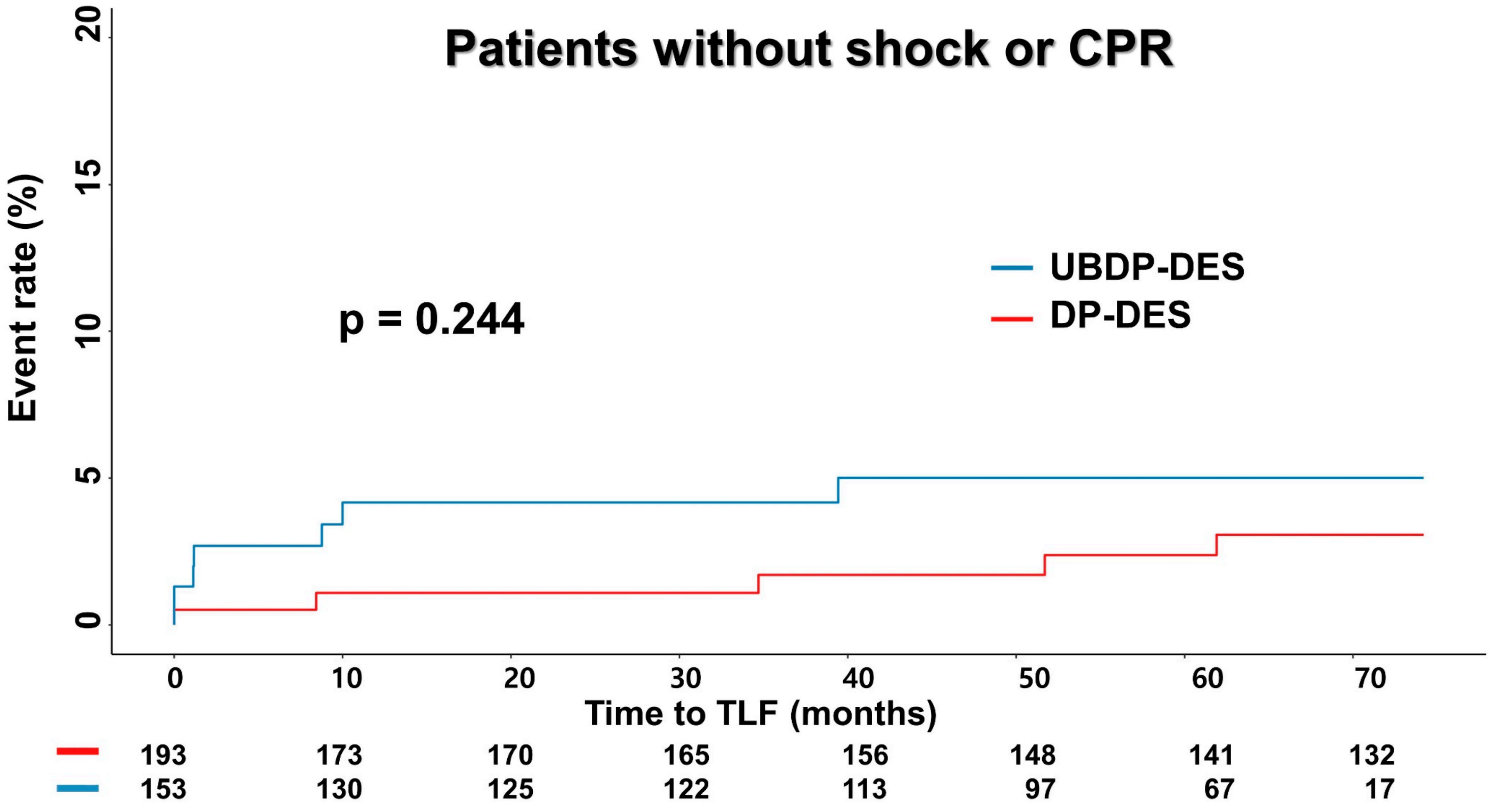

CPR, cardiopulmonary resuscitation.

Other abbreviations are listed in Table 1 and Table 3.

Supplementary Table S1. Clinical characteristics and outcome of patients presenting with Killip class ≥3.

	DP-DES (N = 28)	UBDP-DES (N = 38)	*p*
Male, n (%)	19 (67.9)	31 (81.6)	0.320
Age (years)	64.8 ± 13.3	65.1 ± 11.5	0.926
HTN, n (%)	16 (57.1)	17 (44.7)	0.455
DM, n (%)	13 (46.4)	15 (39.5)	0.754
SBP (mmHg)	124.6 ± 34.7	115.7 ± 43.1	0.357
DBP (mmHg)	80.1 ± 25.2	69.2 ± 26.9	0.095
Heart rate (beats/minute)	80.9 ± 22.0	95.3 ± 30.8	0.030
CPR at initial presentation, n (%)	3 (10.7)	13 (34.2)	0.041
LVEF (%)	44.4 ± 14.4	42.6 ± 15.0	0.623
Initial NT-proBNP (pg/mL [IQR])	7436.2 [2764.6, 14257.3]	3676.4 [885.2, 4176.0]	0.001
Initial CK-MB (ng/mL)	47.8 ± 86.1	33.0 ± 70.9	0.461
Initial Troponin I (ng/mL)	10.7 ± 17.9	7.8 ± 15.5	0.498
Initial hs-CRP (mg/dL)	4.0 ± 5.2	0.8 ± 2.1	0.005
Pre-PCI TIMI ≥ 3, n (%)	2 (7.1)	2 (5.3)	1.000
Post-PCI TIMI ≥ 3, n (%)	24 (85.7)	37 (97.4)	0.154
All-cause mortality, n (%)	4 (14.3)	10 (26.3)	0.381
Cardiac death, n (%)	4 (14.3)	9 (23.7)	0.525
30-day mortality, n (%)	3 (10.7)	6 (15.8)	0.722
TVMI, n (%)	0 (0.0)	0 (0.0)	1.000
ID-TLR, n (%)	1 (3.6)	1 (2.6)	1.000
TLF, n (%)	4 (14.3)	9 (23.7)	0.525

All abbreviations are listed in Tables 1 and 2.
